# Detailed analysis of agro-industrial byproducts/wastes to enable efficient sorting for various agro-industrial applications

**DOI:** 10.1186/s40643-024-00763-7

**Published:** 2024-05-04

**Authors:** Govindegowda Priyanka, Jeevan R. Singiri, Zachor Adler-Agmon, Sasank Sannidhi, Spurthi Daida, Nurit Novoplansky, Gideon Grafi

**Affiliations:** French Associates Institute for Agriculture and Biotechnology of Drylands, Jacob Blaustein Institutes for Desert Research, Ben-Gurion University of the Negev, Midreshet Ben Gurion, 84990 Israel

**Keywords:** Agro-industrial byproducts/waste (AIBW), Wheat bran, Garlic waste, Reuse and recycling, byproduct valorization, waste management, Phytohormones, Weed control, Bacterial growth, strigolactones

## Abstract

Agriculture-based industries generate huge amounts of byproducts/wastes every year, which are not exploited or disposed efficiently posing an environmental problem with implications to human and animal health. Finding strategies to increase the recycling of agro-industrial byproducts/wastes (AIBWs) is a primary objective of the current study. A thorough examination of AIBWs in conjunction with experimental research is proposed to facilitate sorting for various agro-industrial applications and consequently increasing byproduct/waste utilization. Accordingly, two sustainable, locally available sources of AIBWs, namely, wheat bran (WB) and garlic straw and peels (GSP) were studied in detail including content and composition of proteins, phytohormones and nutritional elements, as well as the effect of AIBW extracts on plant and microbial growth. Hundreds of proteins were recovered from AIBW mainly from WBs, including chaperons, metabolite and protein modifying enzymes, and antimicrobial proteins. In-gel assays showed that WB and GSP possess high protease and nuclease activities. Conspicuously, phytohormone analysis of AIBWs revealed the presence of high levels of strigolactones, stimulants of seed germination of root parasitic weeds, as well as indole acetic acid (IAA) and abscisic acid (ABA). Garlic straw extract strongly inhibited germination of the weed *Amaranthus palmeri* but not of *Abutilon theophrasti* and all examined AIBWs significantly affected post-germination growth. Bacterial growth was strongly inhibited by garlic straw, but enhanced by WBs, which can be used at least partly as a bacterial growth medium. Thus, an in-depth examination of AIBW characteristics will enable appropriate sorting for diverse agro-industrial applications, which will increase their utilization and consequently their economic value.

**Supplementary Information:**

The online version contains supplementary material available at 10.1186/s40643-024-00763-7.

## Introduction

Agro-industrial byproducts/wastes (AIBWs) represent a major worldwide problem that is intensified over time as there are no effective ways to treat or utilized the waste (Sadh et al. 2018; Barbera 2020). Most AIBWs are not treated or utilized, and in many cases, AIBWs such as rice straw are often disposed through burning, which leads to an increase in unwanted greenhouse gases (Sadh et al. 2018; Kumar et al. 2023); other AIBW may be disposed through dumping or unplanned landfilling. Improper waste management can lead to severe environmental contamination, which might have a negative impact on both human and animal health and great efforts are being invested worldwide to find efficient solution to reduce, reuse and recycle the ever-increasing AIBWs (Kumar et al. 2023; Sadh et al. 2018). Rice straw can be used for various purposes including biochar production, generation of biofuel as well as a medium for mushroom cultivation (reviewed in Kumar et al. 2023). The potential of using organic waste in Sub-Saharan Africa for energy and biofertilizer production has recently been discussed (Rubagumya et al. 2023).

The wheat grain is defined as caryopsis, a dry, one-seeded indehiscent fruit whereby the seed coat and the pericarp are fused together. Flour production from wheat grains requires milling process in which the outer shell of the grain composed of the pericarp, the testa and the aleurone layers is removed and together with a small fraction of the endosperm attached to them generate the WB. It is estimated that WB comprises of 14.5–25% of the total wheat grain weight (Xie et al. 2008; Neves et al. 2006), and considering that the global wheat utilization forecast for 2023/24, now pegged at 785 million tons (FAO, 2023), WB production will be in the range of 114 to 196 million metric tons (Onipe et al. 2015; Prückler et al. 2014; Reddy and Rhim 2018). Notably, about 10% of the WB byproduct is used in bakeries and in breakfast cereals as a dietary fiber supplement and the remaining bran are channeled to feeding animals or wasted (Xie et al. 2008; Rahman et al. 2017). Indeed, due to high transportation cost, millers often dispose WB as waste, which may cause environmental problems (Rahman et al. 2017). Notably, WB channeled to animal feeding has a low value compared to WB for the food industry (Prückler et al. 2014). Thus, the milling industry is seeking for new approaches for increasing the value of WB particularly as food or food supplement and as a renewable source for extraction of beneficial substances (e.g., proteins, polysaccharides) for various applications (Prückler et al. 2014; Katileviciute et al. 2019). One approach is using WB, as a new source of natural fibers for producing bio-based composites as potential substitutes for petroleum-based materials (Rahman et al. 2021; Safaripour et al. 2021).

Garlic processing wastes, namely, straw and peels (GSP) are produced in large quantities worldwide and have great economic value (Kallel and Ellouz Chaabouni 2017). The global production of garlic in 2020 was about 28 million tons whose processing generates about 3.7 million tons of garlic byproducts/wastes (Sunanta et al. 2023). Most garlic waste is disposed into landfills or burned, which negatively affecting the environment (Kallel and Ellouz Chaabouni 2017). Garlic waste can be used as a source for antimicrobial substances (Naqvi et al. 2020; Singiri et al. 2022), production of cellulose derivatives which can be used as nanofillers for polymer matrices (Moreno et al. 2020; Kallel et al. 2016), as well as for soil amendment and bioenergy (Kallel and Ellouz Chaabouni 2017; Ghani et al. 2019). AIBWs in general can be used for extraction of bioactive substances some of which have beneficial effects on human health including amino acids, phenolics, vitamins, fibers as well as for generating biogas and bio-oil (Sadh et al. 2018; Lopes and Ligabue-Braun 2021; Apprich et al. 2014; Kuparinen et al. 2023).

Despite the large body of research conducted toward valorization of AIBWs, the research addressing the potential reuse and recycling of these wastes including WB and GSP is insufficient. Consequently, reuse and recycling of these byproducts/wastes are very limited, and the vast majority is still disposed inappropriately by dumping, landfilling, and burning (Wilson et al. 2015; Kaza et al. 2018); their quantities are steadily increasing every year. It is suggested that a thorough understanding of AIBW characteristics, such as their proteome and metabolome, would enable more effective sorting for a range of agro-industrial uses, thereby increasing their utilization and consequently their economic value. Focusing on two locally available AIBWs that are produced in large amounts worldwide, namely, WB and GSP revealed multiple beneficial substances accumulated in these AIBWs including proteins (proteomics), phytohormones and nutritional elements. Bioassay analysis uncovered the potential of WB and GSP to provide beneficial substances for direct use in agriculture to support and enhance growth of crop plants and combat potential weeds as well as providing substances that can enhance or inhibit bacterial growth.

## Materials and methods

### AIBWs and their extraction

WB was obtained from a local flour mill ‘Stibel’ located at Beer Sheva, Israel. We obtained four different batches of WB designated WB-1, WB-2, WB-3, and WB-4 (supplementary Fig. S1). Each batch was analyzed separately, keeping the authentic way the company collects WB into big containers without recording the exact sources of cereals in each batch. Garlic processing waste straw (Gs) and peel (Gp) were obtained from a garlic processing company Dorot, Israel. Extraction of AIBWs was performed by incubating grounded AIBW in phosphate-buffered saline (PBS, 1:10 w/v), except for germination experiments where AIBWs were extracted with deionized H_2_O, at 4^o^C for 16 h, with gentle shaking, followed by high-speed centrifugation (16,000 x g, 4^o^C). The cleared supernatant was collected and stored at -20^o^C until used.

### Proteome analysis

Proteomic analysis of WB and GSP was performed by the proteomic services of The Smoler Protein Research Center at the Technion, Haifa, Israel. Proteins released from 10 mg of dry waste incubated in 100 µL PBS at 4^o^C for 16 h with gentle agitation were digested with trypsin, followed by separation and mass measurement via liquid chromatography with tandem mass spectrometry (LC-MS/MS) on LTQOrbitrap; ThermoFisher Scientific, Waltham, MA, USA). Protein identification and quantification were done using MaxQuant, using *Brachypodium distachyon* proteins from Uniport as a reference. All the identified peptides were filtered with high confidence, top rank, and mass accuracy. High-confidence peptides passed the 1% FDR threshold (FDR = false discovery rate, the estimated fraction of false positives in a list of peptides). Three replicates were performed for each examined waste. A protein was considered “present” if it occurred in at least two replicates and it is represented by at least 2 peptides. GO categorization analysis was carried out using the PANTHER classification system (Mi et al. 2021) against the *Triticum aestivum* UniProt database and the following GO trees were examined: biological process and protein class.

### In-gel nuclease assay

Nuclease assay was performed essentially as described (Blank et al. 1982) in polyacrylamide gel containing 300 µg/mL denatured salmon sperm DNA. Extract equivalent to 2.5 mg of dry AIBW was incubated with sample buffer for 1 h at 37^o^C followed by separation on 12% sodium dodecyl sulfate polyacrylamide gel electrophoresis (SDS-PAGE). The gel was washed twice (30 min. each) at room temperature in buffer containing 10 mM Tris-HCl pH 7.5 and 25% isopropanol, followed by washing twice, 15 min each, with 10 mM Tris-HCl pH 7.5. Nuclease activity was performed by incubating the gel with 10 mM Tris-HCl pH 7.5 containing divalent cations (10 mM MgSO_4_, 10 mM CaCl_2_) for 75 min at 37^o^C. The gel was stained for 60–80 min with 10 mM Tris HCl pH 7.5 containing 2 µg/mL ethidium bromide and inspected under ultra-violet (UV) light.

### In-gel protease assay

In-gel protease assay was performed essentially as described (Solomon et al. 1999). Samples equivalent to 2.5 mg of dry AIBW were loaded and run in 12% SDS-PAGE containing 0.12% gelatin. After running was completed, the gel was washed twice, 45 min each, in buffer containing 10 mM Tris-HCl (pH 7.5) and 0.25% Triton x-100 followed by overnight incubation in 10 mM Tris-HCl (pH 7.5). The gel was then incubated in 10 mM Tris-HCl (pH 7.5) containing 10 mM CaCl_2_ and 10 mM MgCl_2_ for 30 min at 30^o^C and stained by Coomassie blue for 1 h at room temperature.

### Phytohormones analysis

Phytohormones in selected AIBWs, namely, WB-1, Gs and Gp was analyzed by Creative proteomics (NY, USA). About 25 mg of AIBW samples were used for extracting and analyzing phytohormones using a targeted LC MS/MS workflow. A 15 µL aliquot was diluted two times with MQ-water and transferred into the HPLC vials, ABA, SA, JA, JA-Ile, OPDA, IAA, IAA-Asp, IAA-Ala, IAA-Trp, Methyl IAA, c-and t-zeatin, t-zeatin riboside, strigol and, DIMBOA were separated using a ZORBAX Eclipse Plus C18 column (2.1 × 100 mm, Agilent) running at a flow rate of 0.45 mL/min. The data was normalized based on internal standards D6ABA, D5IAA, D4SA, D2JA, D2GA1 to account for experimental variation and hormone extraction/ionization efficiency.

### Analysis of nutritional elements

Grounded AIBWs (30 mg) were incubated with 600 µl Milli-Q for 16 h on an orbital shaker at 4 °C. After incubation samples were centrifuged at high speed (16,000 x g) and the supernatant was collected, filtered through 0.22 μm spin filter and 200 µl of each sample were diluted with 5.8 ml of Milli-Q water and subjected to micro and macro-element analysis by the inductively coupled plasma-optical emission spectroscopy (ICP-OES) using ICP-720-ES (Varian Inc., USA).

### Germination assays

Experiments were conducted to assess the impact of WB and GSP extracts (in water) on the germination and post-germination growth of the weeds *Abutilon theophrasti* (3 replicates, 10 seeds / replicate) and *Amaranthus palmeri* (4 replicates, 25 seeds / replicate). These experiments were carried out in petri dishes on blot paper, supplemented with water or with extracts of WBs and GSP, in a growth room in the dark under temperature of 22 °C; germination was monitored at 24, 48, and 72 h after sowing.

### Bacterial growth assay

The assay was performed essentially as described (Patton et al. 2006). Briefly, *Staphylococcus aureus* was grown overnight in LB medium at 37◦C, the culture was diluted, transferred to Luria Broth (LB) medium and grown at 37◦C to 0.03–0.05 optical density (OD595; Epoch, Biotek, Winooski, VT, USA). To a 150 µL aliquot of the culture, 50 µL of LB (control 1), H_2_O (control 2), kanamycin (final concentration 50 µg/ml), and 50 µL of AIBW extracts sterilized by passing through 0.2 μm filter were added (six replicates per treatment) in a flat-bottom 96-well microtiter plate. Plates were incubated in the dark using a spectrophotometer (Synergy 4, Biotek, USA) and reads (OD595) were taken in intervals of 30 min in a course of 12 h. The average OD for each blank replicate at a given time point was subtracted from the OD of each replicate treatment at the corresponding time point and standard errors were calculated for each treatment at every time point. In a complementary experiment, we analyzed the capacity of WB-1 to serve as a substitute for the bacterial growth medium. Briefly, WB-1 extract (10 g/50 ml) of various dilutions (100% WB-1, 75% WB-1, 50% WB-1) along with LB was used to analyze the growth of *S.aureus* in a flat-bottom 96-well microtiter plates. Each treatment was performed in seven or eight replicates. This experiment was conducted twice.

### Statistical analysis

Unpaired t test was performed using the GraphPad QuickCalcs Web site: https://www.graph.

pad.com/quickcalcs/ttest1/?Format = C (accessed November 2019) or using the Microsoft Excel platform. For comparison of multiple groups we used one-way ANOVA calculator, with Tukey HSD at confidance level of 95% (https://www.socscistatistics.com/tests/anova/default2.aspx).

## Results

### Proteome analysis of AIBWs

**Fig. 1:**
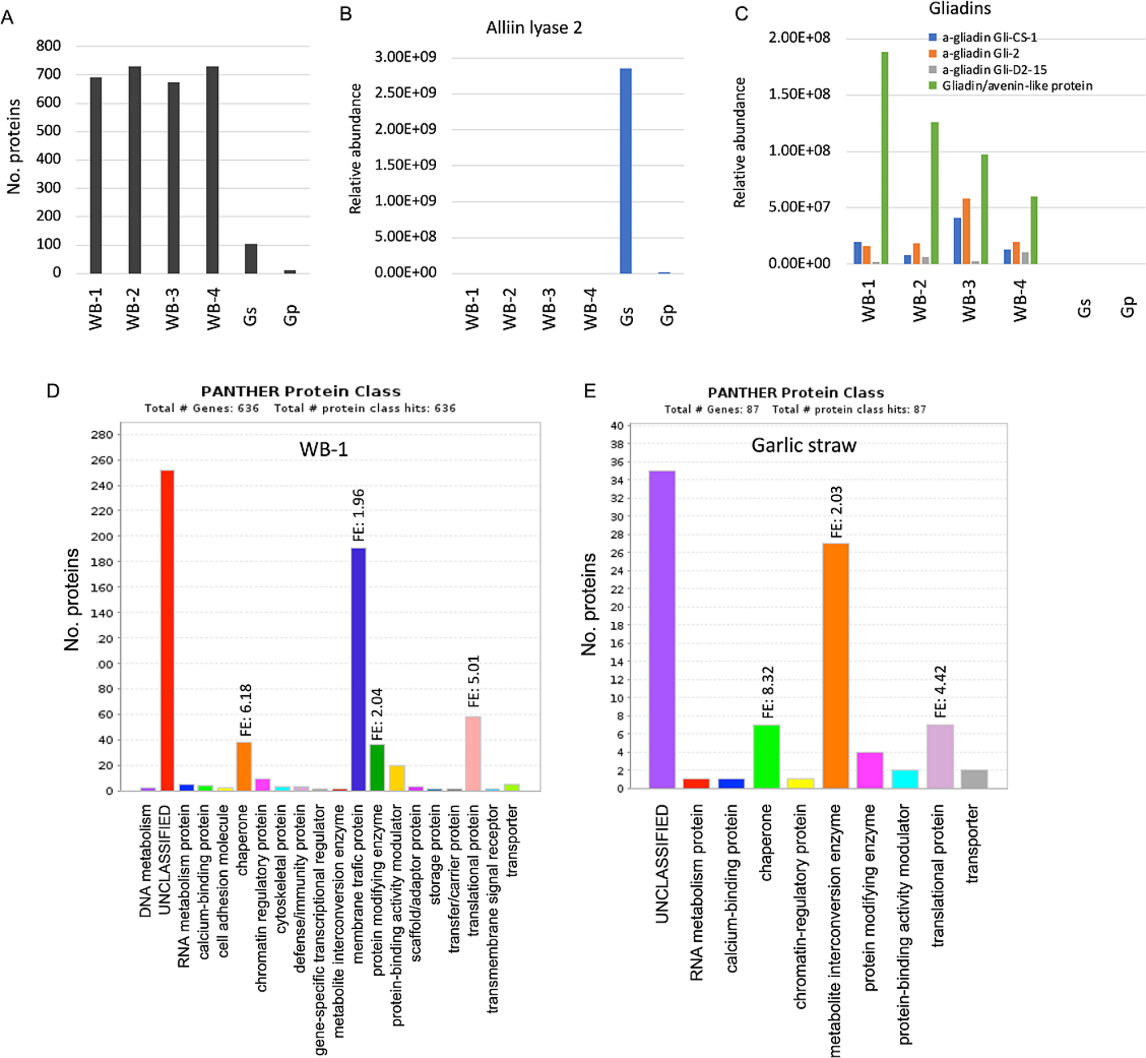
Proteome data analysis. (**A**) The number of proteins identified in the indicated AIBWs. A protein was considered present if it is represented by at least 2 peptides and displays non-zero values in at least 2 repeats. WB, wheat bran; Gs, garlic straw; Gp, garlic peel. (**B**) Alliin lyase 2 (alliinase) is exclusively present in GSP. (**C**) The wheat storage proteins gliadins are recovered solely from WB. (**D**) Protein class categorization of protein identified in WB-1. (**E**) Protein class categorization of protein identified in Gs. Fold enrichment (FE) is shown (FDR < 0.05, PANTHER classification system)

**Fig. 2:**
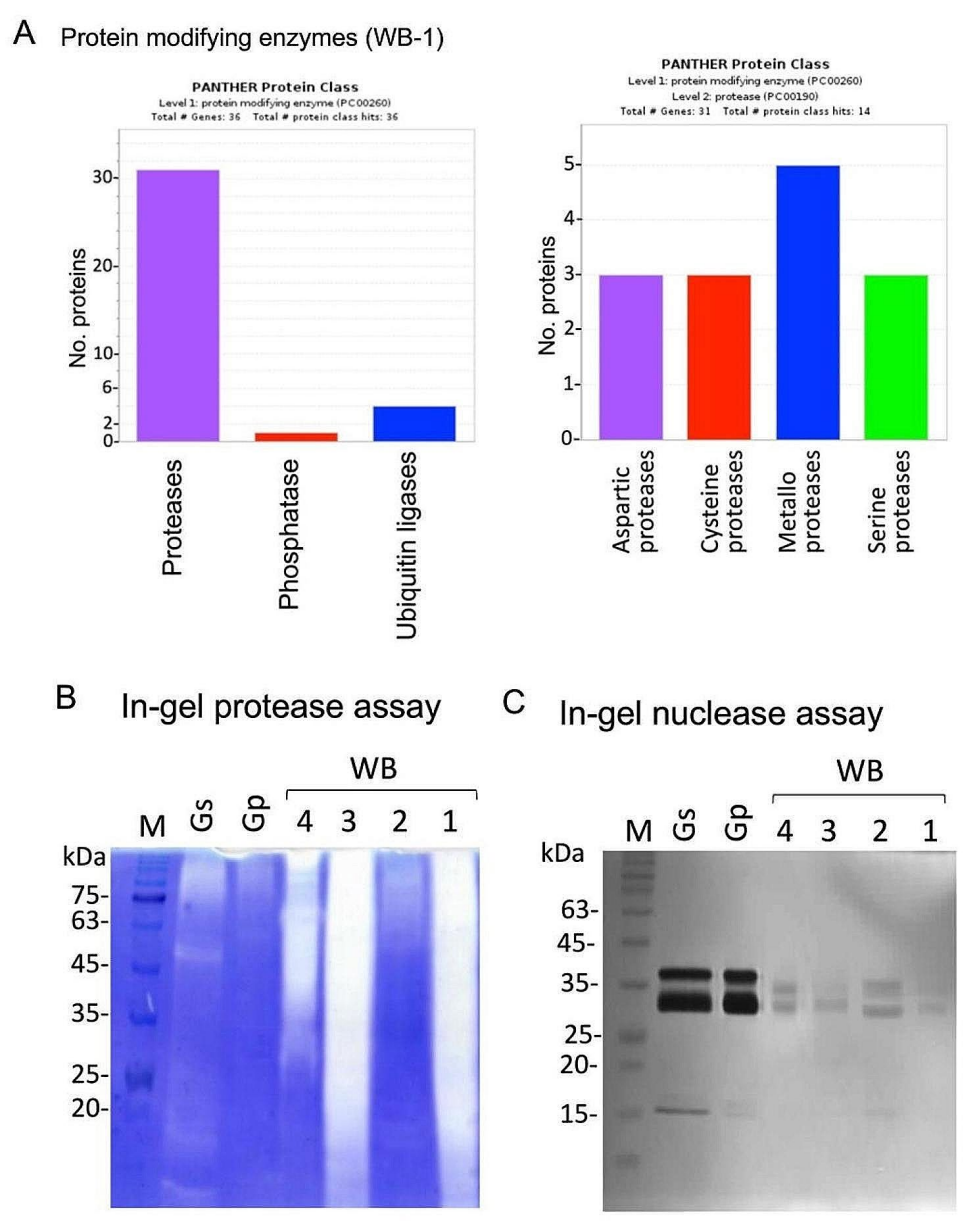
Categorization of protein modifying enzymes identified in WB-1 (**A**). Note that most modifying enzymes are proteases (left panel) of various classes (right panel). In gel activity assays for proteases (**B**) and nucleases (**C**) in the indicated AIBWs, namely, garlic straw (Gs), Garlic peel (Gp) and wheat bran (WB-1 to WB-4). Note, the notable smear appearance of proteases in WB is indicative for SDS-resistant proteases. M, protein weight markers in kilodalton (kDa)

Proteome analysis was performed on AIBWs derived from wheat milling (four different batches of bran WB-1 to WB-4) and garlic processing wastes (GSP), straw (Gs) and peel (Gp). Notably, each batch of WB was analyzed separately, keeping the authentic way of collecting WB by the milling company. Proteome analysis was performed by using LC-MS/MS on LTQ-Orbitrap and identification by Discoverer software against the *Triticum* from Uniprot databases. The raw and the initial filtered data are shown in supporting data Table S1 and S2. This analysis uncovered several hundreds of proteins having at least 2 peptides, most were extracted from WBs (∼ 700 proteins) and the least from Gs (100 proteins) and Gp (11 proteins) (Fig. 1A, see also supporting data Table S3-S8). The protein data showed that the enzyme alliin lyase 2 also known as alliinase responsible for converting alliin into alliicin, a compound that provides fresh garlic its signature aroma (Rabinkov et al. 1994) is detected only in garlic, mainly in Gs (Fig. 1B); the wheat storage proteins, gliadins were recovered from WB only (Fig. 1C). Notably, gliadins are efficiently extracted in alcohol to yield a fraction which is rich in gliadins, yet low level of gliadins can be extracted in aqueous solution like PBS (Güven and Azizoglu 2023). Protein class categorization of the proteins identified in WB-1 and in Gs highlighted metabolite and protein modifying enzymes, chaperones, and translational proteins which appear to be overrepresented in WB-1 and Gs (Fig. 1D and E). Among the 34 modifying enzymes listed in WB-1, 31 are proteases and include aspartic proteases, serine proteases, cysteine proteases and metalloproteases (Fig. 2A). Metabolite modifying enzymes are particularly enriched in oxireductases both in WB-1 (supplementary Fig. S2A) and garlic straw (Fig. S2B). Reactive oxygen species (ROS) detoxifying proteins were identified mainly in WB including peroxidases, catalases, and superoxide dismutases (Fig. S3A) while defensins, small anti-fungal proteins are abundant in WB but not in GSP (Fig. S3B). Other anti-microbial proteins include chitinases, which are abundant in WB and the agglutinin isolectin 1, which is enriched in Gp (Fig. S3C). Finally, categorization for biological process (Supplementary Fig. S4) showed that proteins implicated in response to stress are overrepresented in WB-1 and Gs.

### Hydrolytic enzyme activities in AIBWs

The finding that AIBWs, particularly WB are rich in proteases prompted us to analyzed hydrolytic enzyme activities using in-gel protease and nuclease assays. In-gel protease assay revealed that both WB and GSP possess notable protease activities. A high level of protease activity was found (Fig. 2B) in WB-1 and WB-3, and their smear appearance suggests some proteases are likely to be SDS-resistant (Blumentals et al. 1990). In-gel nuclease assay showed that GSP and WB possess notable levels of nucleases migrating in the gel to positions of about 35 kDa and low level of nucleases migrating to position of about 15 kDa (Fig. 2C). Thus, hydrolytic enzymes retain their activity in dry, essentially dead organs that constitute the examined AIBWs.

### Analysis of phytohormones

The presence of phytohormones and related bio-stimulants was analyzed in selected AIBWs, namely, WB-1, Gs and Gp. Analysis was performed by Creative Proteomics (NY, USA) using a targeted LC MS/MS workflow. The phytohormones IAA-Ala, 5-deoxy-strigol (5DSTR), the benzoxazinoid DIMBOA, and the gibberellic acids, GA1, GA3, GA4, GA8, GA9, GA20, GA29 in all samples analyzed were below the lower limit of quantification. Other phytohormones including abscisic acid (ABA), salicylic acid (SA), jasmonic acid (JA), JA-Ile, 12-oxophytodienoic acid (OPDA), IAA, IAA-Asp, IAA-Trp, Methyl IAA, c-and t-zeatin (cZ and tZ), c- and t-zeatin riboside (cZR and tZR), strigol and orobanchol as well as the gibberellic acids GA12, GA19 and GA53 were identified in the AIBW samples.

The results showed (Table 1) that the examined AIBWs, each possesses various phytohormones at different levels. Thus, Gp contains extremely high levels of strigol (∼ 164 µg/gDW), Gs has 4.375 µg/gDW and none in WB-1. On the other hand, while WB-1 contains high level of orobanchol (492 ng/gDW), none is detected in Gs and Gp. Both strigol and orobanchol are strigolactones (SLs) that were initially characterized as stimulants of seed germination of species in the genera Striga, Orobanche, and Phelipanche, a group of root parasitic weeds of global economic importance (Parker 2012). In WB-1, IAA, IAA-Asp and SA were detected at a relatively high level, while gibberellic acids and cytokinins were at a relatively low level (Table 1). IAA was particularly high in Gs (1.65 µg/gDW), and at a moderate level in Gp (257 ng/gDW) and in WB-1 (367 ng/gDW). ABA, a stress phytohormone, was not detected in WB-1 but in Gs (323 ng/gDW) and Gp (318 ng/gDW). Thus, WB and GSP appear to be rich in certain growth factors that could affect plant growth and development.

**Table 1:** Phytohormones (PhytH) and related compounds detected in the examined AIBWs

AIBW	WB-1	Garlic straw	Garlic peel
PhytH	ng/gDW		
ABA	0.00	323.1	318.3
SA	224.9	63.3	104.5
JA	26.6	44.7	41.4
JA-ILE	1.9	16.9	24.7
OPDA	6.2	11.0	30.6
IAA	367	1651	257.4
IAA-Asp	238	45.5	61.9
IAA-Trp	6.1	3.1	14.8
MethylIAA	3.0	0.9	2.6
Orobancol	492	0.0	0.00
Strigol	0.0	4375.5	163,905
GA12	0.0	17.3	2.9
GA19	14.6	0.0	0.00
GA24	0.0	0.0	0.00
GA53	10.7	9.1	11
cZ	0.8	1.6	0.3
cZR	7.6	5.4	4.1
tZ	9.1	1.3	2.8
tZR	3.6	1.9	2.04

ABA, abscisic acid; SA, salicylic acid; JA, jasmonic acid, JA-ILE, JA-isoleucine; OPDA, 12-oxophytodienoic acid; IAA, indole acetic acid; IAA-ASP, IAA-aspartate, IAA-Trp, IAA tryptophane; Methyl IAA, cZ, cis-Zeatin; tZ, trans-Zeatin; cZR, cis-Zeatin riboside; tZR, trans-Zeatin ribosode; GA, gibberellic acids

### Analysis of nutritional elements

**Fig. 3:**
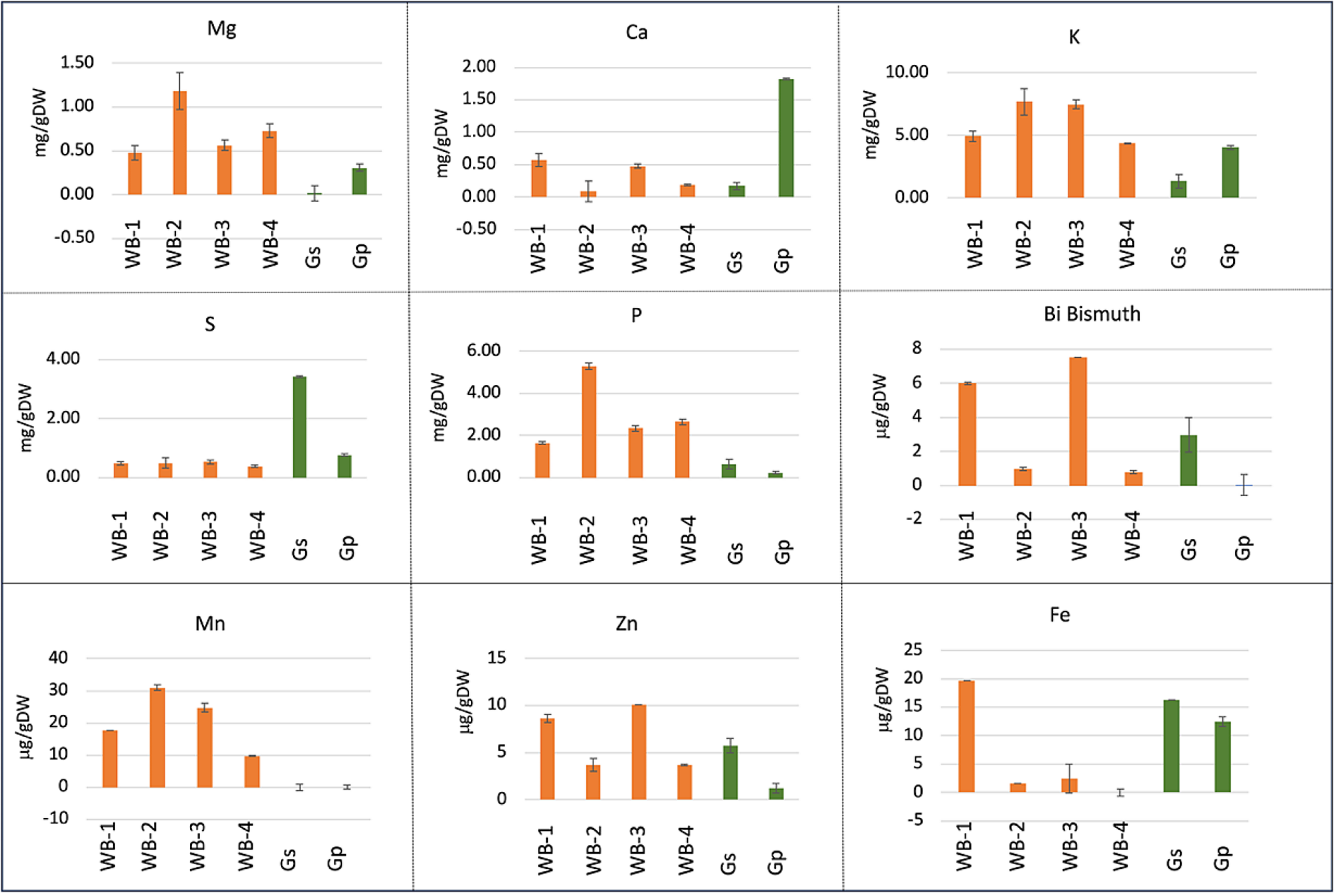
Nutrient levels in AIBWs. AIBWs extracts were subjected to nutrient detection by ICP-OES. The concentration of each macroelement (Mg, magnesium; K, potassium; Ca, calcium; S, sulfur; P, phosphorous) is given in milligram (mg) per gram dry weight (gDW) and that of microelements (Mn, manganese; Zn, zinc; Fe, iron; Bi, Bismuth) in microgram (µg)/gDW. Vertical bars represent the standard deviation (*n* = 3)

Ground AIBWs were extracted with water and subjected to micro and microelement analysis. The results (Fig. 3) show variability between AIBWs with respect to nutritional element composition and levels. Generally, WBs were found enriched with magnesium (Mg), potassium (K) and phosphorous (P), while garlic wastes possess high levels of calcium (Ca) and sulfur (S). The microelement manganese (Mn) was barely detected in garlic but was relatively high in WBs, zinc (Zn) and Bi (Bismuth) were relatively high in WB-1, WB-3 and Gs, and iron (Fe) was high in WB-1 and GSPs (Fig. 3).

### Effects of AIBW extracts on germination and post-germination growth of the weed amaranthus palmeri

**Fig. 4:**
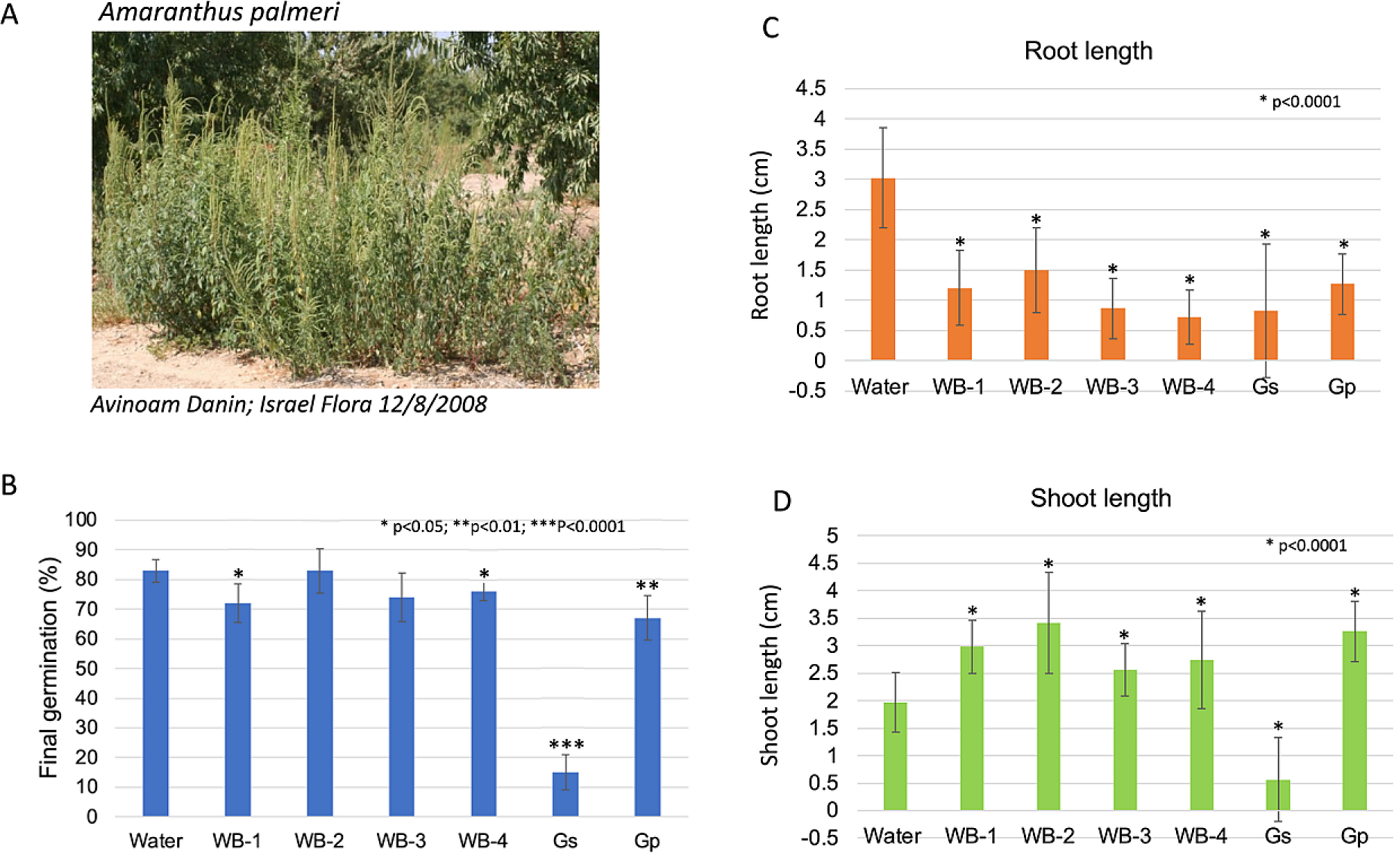
Effect of AIBW extracts on germination and post germination growth of the weed *Amaranthus palmeri*. (**A**) *A. palmeri* plants in orchard, from Israel Flora with permission. (**B**) Seeds of *A. palmeri* were germinated in water or in extracts derived from the indicated wastes and germination percentage was recorded after 72 h. Vertical bars represent the standard deviation. Germinated seedlings were inspected 5 days after sowing and root (**C**) and shoot (**D**) lengths were recorded, and each treatment was compared to water. Vertical bars represent the standard deviation. Statistically significant differences between water and the examined AIBW are indicated by asterisks. The *p* value was determined using Student’s unpaired t-test (GraphPad software)

The weed *A. palmeri* is a fast-growing C4 plant (Fig. 4A), which significantly reduced growth and yield of multiple crops including corn, cotton, soybean, and peanut (Sarangi et al. 2021). Thus, the potential of AIBWs to control germination and post germination growth of this weed was addressed. Preliminary germination experiments showed that *A. palmeri* seeds germinated at a level of 86% within 72 h. Seeds of *A. palmeri* were germinated in water or in extracts derived from WB and GSPs. As shown in Fig. 4B, WB-1, WB-4 and Gp extracts slightly reduced germination of *A. palmeri*; the strongest inhibitory effect was obtained under Gs extract (15% germination). Notably, all extracts significantly affected post germination growth of *A. palmeri* seedlings. Accordingly, all examined extracts reduced root growth compared to water (Fig. 4C), while increasing shoot length except for Gs where the shoot length was substantially reduced (Fig. 4D). Thus, it appears that while extract of Gs has a very strong allelopathic activity against *A. palmeri* germination, all other examined AIBW extracts mainly affected post germination growth with a notable effect on root length.

### Effects of AIBW extracts on germination and post-germination growth of the weed Abutilon theophrasti

**Fig. 5:**
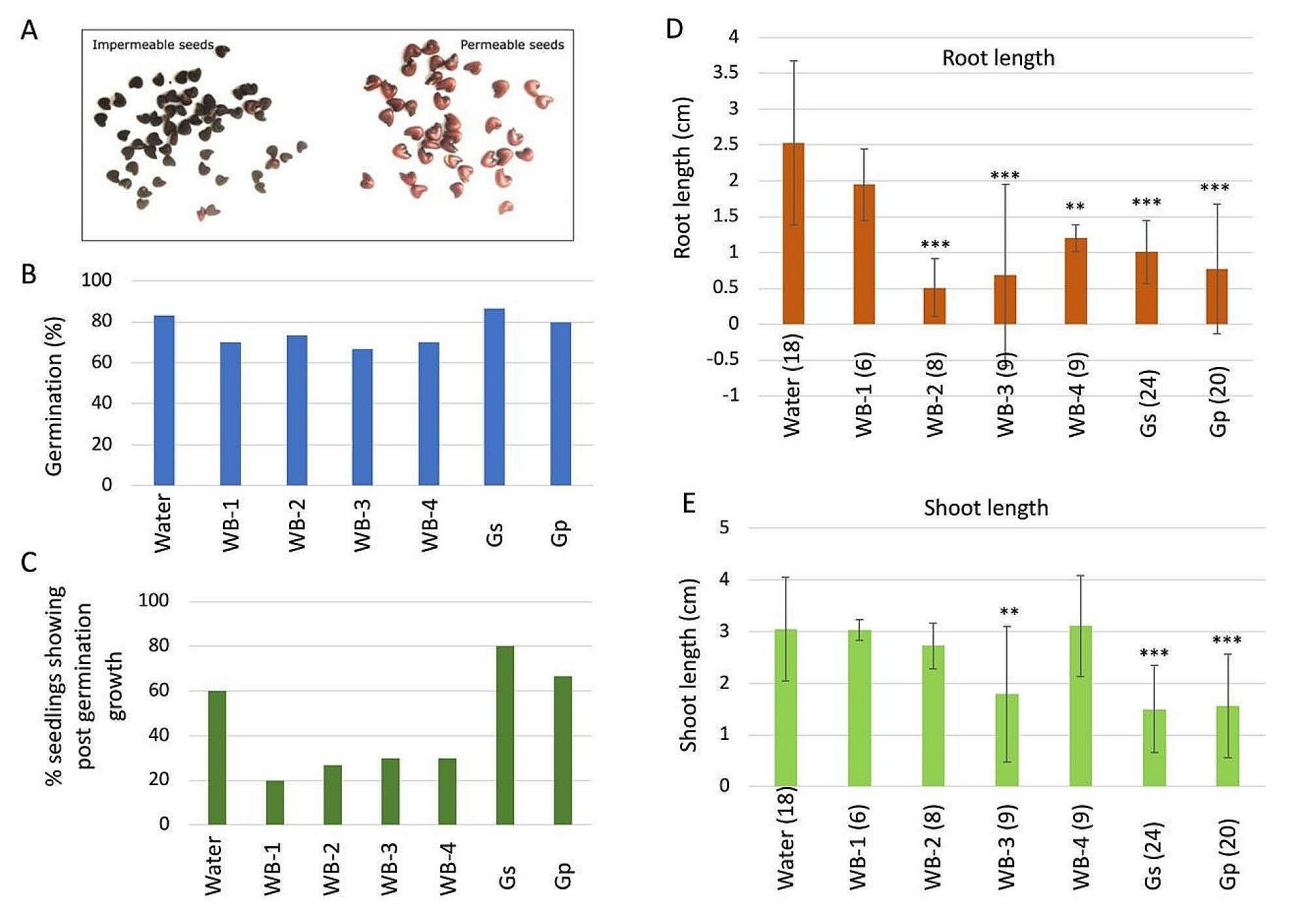
Effect of AIBW extracts on germination of the weed *Abutilon theophrasti*. (**A**) Seed imbibition of *A. theophrasti.* Seeds imbibed in water for 24 h at 37^o^C displayed two types of seeds, water impermeable (left panel) and permeable (right panel). (**B**) Seeds were germinated (3 repeats, each 10 seeds) in water or in extracts derived from the indicated AIBW and germination percentage was recorded after 72 h. Asterisk indicates statistically significant difference between AIBW and water (*p* < 0.05) (**C**) Percentage of seedlings showing post germination growth. (**D**) Effect of AIBWs on shoot growth. (**E**) Effect of AIBWs on root growth. Each treatment was performed in 3 replicates, each containing 10 seeds. Vertical bars represent the standard deviation. Numbers in brackets (panel **D** and **E**) are the number of seedlings showing post germination growth. Asterisks indicate the different levels of significance between AIBW extracts and water treatments (**, *p* < 0.01; ***, *p* < 0.001). The *p* value was determined using Student’s unpaired t-test (GraphPad software)

*Abutilon theophrasti* is a common problematic weed in agricultural fields which spreads via abundant seeds that can persist in the soil for many years. It is showing resilient against allelochemicals and causing large economic losses (Tabaglio et al. 2023). Preliminary germination experiments showed that *A. theophrasti* has two types of seeds that can be distinguished following imbibition, about 50% of the seeds imbibed water, swelled, and attained brown color and 50% are water-impermeable and remain black (Fig. 5A); water-permeable seeds were selected for further germination assays. Imbibed seeds were incubated in water or in AIBW extracts derived from WB and GSPs. As shown in Fig. 5B, seed germination was slightly reduced though showing significant difference between germination in water and germination in extracts derived from WB-1, WB-3 and WB-4. Post germination growth was markedly affected by AIBW extracts, with negative effect under extracts derived from WBs in which post germination growth of most seedlings was halted (Fig. 5C). Although, the proportion of seedlings showing post germination growth was higher under GSPs, the root and shoot growth were significantly decreased compared to water (Fig. 5D and E). All AIBW extracts, except for WB-1, had a negative effect on root growth of *Abutilon* seedlings (Fig. 5D).

### Effects of AIBW extracts on microbial growth

**Fig. 6:**
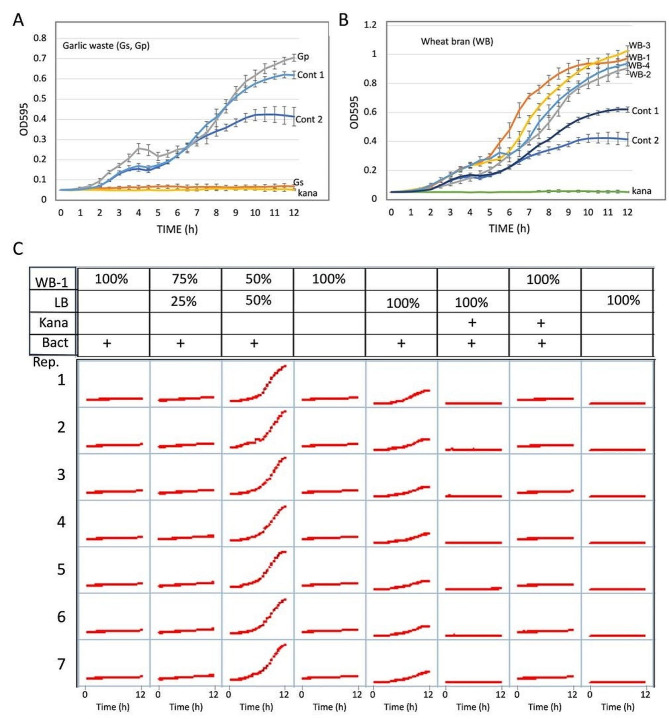
WB and GSP contain microbial growth controlling substances. *Staphylococcus aureus* bacterial culture at 0.05 OD_595_ (150 µl) was grown in a flat-bottom 96-well microtiter plate supplemented with 50 µl of LB (cont 1), H_2_O (Cont 2) or AIBW extracts derived from garlic straw (Gs) and garlic peel (Gp) (**A**) or from wheat bran (WB-1 to WB-4) (**B**). Kanamycin (Kana) was used as an antibiotic reference. (**C**) WB-1 extract can serve as a bacterial growth medium to enhance bacterial growth. *S. aureus* (10 µl of overnight culture) was grown in a flat-bottom 96-well microtiter plate in the presence of various concentrations of WB-1 extract diluted with LB and compared to LB (100%). Bacterial growth experiments were performed in seven replicates each treatment, and vertical bars in A and B represent the standard deviation. Kanamycin (Kana) was used as an antibiotic reference. Bacterial growth (OD_595_) was measured at 0.5 h intervals in the course of 12 h

The effect of WB and GSP extracts on the growth of the Gram positive bacteria *Staphylococcus aureus* was examined. To this end, bacteria were grown in a flat-bottom 96-well microtiter plate in LB medium supplemented with Gs and Gp extracts (Fig. 6A), or with extracts derived from WB-1, WB-2, WB-3 and WB-4 (Fig. 6B). The LB medium (Control 1) and H_2_O (Control 2) were used as controls; kanamycin was used as antibiotic for *S. aureus*. Plates were incubated in the dark using a Synergy 4 spectrophotometer (Biotek, Winooski, VT, USA), and reads (OD 595) were taken at 30 min intervals over a course of 12 h. A strong inhibitory effect was obtained with Gs which is comparable to the effect of kanamycin (Fig. 6A). All other extracts examined, namely Gp and particularly WBs significantly promoted bacterial growth (Fig. 6A, B). Finally, we investigated the capacity of WB-1 to serve as a substitute for the bacterial growth medium. Various dilution of WB-1 extracts (10 g/50 ml water) with the Luria Broth (LB) standard medium (100% WB-1, 75% WB-1, 50% WB-1 and 100% LB) were used and growth of *S*. *aureus* was analyzed in a flat-bottom 96-well microtiter plate. The results showed (Fig. 6C and supplementary Fig. S5) that bacterial growth was strongly enhanced under 50% WB-1 extract compared to 100% LB. Higher concentration of WB-1 (100% and 75%) inhibited growth similarly to kanamycin.

## Discussion

The data presented here highlighted AIBWs, namely the WB and GSP as sustainable, rich sources for beneficial substances that can be exploited for use in agriculture as well as in the industry for various purposes including stimulating plant growth, protection against potential pathogen, combating weeds as well as in the food- and non-food industries. Notably, the WB analyzed in the present study mostly represent dead organs that enclosed the embryo, namely, pericarp, seed coat and endosperm contaminant but also live cells embedded within broken seeds, while GSP represent dry garlic residues (garlic straw) and the dead outer and inner garlic peels (Gp) enclosing the vegetative reproductive structure (Singiri et al. 2022). The abovementioned organs undergo programed cell death (PCD) upon maturation, a process where macromolecules such as proteins, RNAs and DNAs undergo degradation by multiple hydrolytic enzymes (DNAses, RNases, proteases) and their constituents remobilized to other plant parts for supporting growth or storage. Yet, previous reports have demonstrated that during PCD hundreds of proteins remain intact, and are stored within the dead organs, and retain their enzymatic activities for decades (reviewed in Grafi 2020; Raviv et al. 2018).

### Beneficial substances in AIBWs

Both WB and GSP store multiple nucleases and proteases that retain high hydrolytic activities. These nucleases and proteases were probably engaged in degrading DNA, RNA, and proteins during PCD at the time of maturation of wheat grains (caryopses) and garlic bulbs. These results are consistent with previous reports demonstrating the release of nucleases and proteases from garlic inner and outer peels (Singiri et al. 2022) and from caryopsis and dead floral bracts of various wheat cultivars (Raviv et al. 2017). It should be noted that different batches of WB display different properties, which might be attributed to the cereal varieties processed, stage of milling as well as to maternal environment (Khadka et al. 2020; Raviv et al. 2020; Swetha et al. 2021; Singiri et al. 2021).

Proteases are important factors in biotechnological applications and especially in increasing the value (valorization) of byproducts such as whey, feathers, collagen, and fish and soy proteins (Troncoso et al. 2022). The activity of the proteases allows the formation of active biopeptides, which are products of high added value with many beneficial effects on human health (Troncoso et al. 2022). The common source of proteases is of microbial origin, but their production cost is very high. Plant proteases are similarly effective and sometimes show better activity than animal and microbial proteases (Troncoso et al. 2022). Traditional plant sources for proteases for industrial use have a relatively high cost and include fruits, roots, and leaves of papaya (papain), stems and juices of pineapple (bromelain), milky resin of the fig (picin) as well as kiwi fruit (actinidin) (Troncoso et al. 2022). This is in addition to the need to allocate designated agricultural areas for growing plants to produce proteases. The data presented here highlight AIBWs as rich, sustainable sources for proteases that can be utilized. Some of the SDS-resistant proteases found in WB could be used as detergent additives in laundry and washing dish machines (Zhang et al. 2022), while others can be used in the food industry for meat tenderization, degradation of allergenic proteins (e.g., gluten) and for production of biopeptides (Troncoso et al. 2022). Thus, utilization of AIBWs for production of proteolytic enzymes for multiple purposes offers many advantages, including reduced labor and material costs, as well as environmental advantages such as decreased effluent output and reduction of the AIBW load.

WB but not GSP released several defensin-like (DEFL) proteins also known as low molecular weight cysteine-rich (LCR) proteins, previously described in WB (Balandrán-Quintana and Mendoza-Wilson 2018) and were shown to act against potential fungal pathogen (Carvalho and Gomes 2011; De Coninck et al. 2013). Defensins were initially found in seeds, but they are also present in leaves and flowers, and often these genes are activated following exposure to biotic and abiotic stress conditions (Vriens et al. 2014) and could enhance resistance to pathogen in transgenic plants (Coca et al. 2004). Other proteins listed in the proteome data that could act against pathogens include chitinases, endochitinases, endonucleases and glucanases. Chitinases are enzymes that break down chitin, an abundant polysaccharide and a major component of cell walls in fungi. Genes encoding for chitinases and glucanases were often over-expressed in plants to achieve resistance against fungal pathogens (reviewed in Ceasar and Ignacimuthu 2012). Finally, waste and byproducts derived from *Solanum tuberosum* and Brassicaceae species appear to be rich sources for bioactive compounds such as glycoalkaloids and glucosinolate hydrolysis products that can protect crop plants against various biotic stresses including fungi and insects (Pacifico et al. 2021).

### Phytohormones

The analysis of phytohormones revealed some striking finding not described previously, namely, the presence of orobanchol and strigol at high levels in WB and GSP. Both are strigolectones known to stimulate seed germination of root parasitic weeds in the genera Striga, Orobanche, and Phelipanche. They cause significant losses of yield as they adapt rapidly to new resistant crop cultivars and traditional herbicides are ineffective (Parker 2012; Yoder and Scholes 2010). Multiple studies suggested consistent reductions in parasitic weed seed bank in infected fields by applying intercropping and crop rotation. Thus, selection of non-host crops with high production levels of strigolactones like garlic and wheat for intercropping and crop rotation may be advantageous in reducing the seed load of root parasitic weeds in infected fields. It would be important to analyze the levels of strigolactones in additional non-host crop plant residues for efficient intercropping and crop rotation implementation in various agroecosystems infected in root parasitic weeds.

Besides strigolactones, WB and GSP possess certain phytohormones including IAA, ABA and salicylic acid (SA) at relatively high levels; garlic straw contains the highest level of IAA (1.65 µg/gDW). Accordingly, extraction of 10 mg dry weight of Gs in 100 µl of buffer will give rise to about 0.94 µM of IAA in the extract, a concentration shown to enhance lateral root formation on primary root of *Arabidopsis* (Oono et al. 2003). The IAA conjugate IAA-Asp, which is thought to be the starting point in IAA catabolism (Ludwig-Müller 2011) is relatively high in WB-1 but very low in Gs/Gp wastes. SA is a well-known phytohormone that plays key roles in plant immunity (Koo et al. 2020) and together with other phytohormones like ABA, IAA and ethylene are commonly used in seed priming to enhance seed performance and fate particularly under stress conditions (Marthandan et al. 2020; Rhaman et al. 2021). Notably, plant residues have been reported to exert priming activity on various plant species. Accordingly, seeds of *Camelina sativa* primed with dried sorghum water extracts showed improved performance under salt stress (Huang et al. 2021). Similarly, seed priming of peanut, okra, sunflower, and chickpea with extracts of dried leaves of *Acacia nilotica* and *Sapindus mukoross* showed a significant reduction in root rot infection caused by *Rhizoctonia solani, Fusarium spp.* and *Macrophomina phaseolina* (Rafi et al. 2015). On the other hand, *Eucalyptus camaldulensis* dried leaf extract had allelopathic effect against germination, vigor and growth rate of various crop plants (Ahmed et al. 2008).

### Allelopathy

AIBW extracts also accumulate allelopathic substances that selectively inhibit or promote seed germination and post germination growth. Often dead organs enclosing the embryo such as the seed coat, the pericarp and floral bract in grasses (i.e., glume, lemma, paleae) as well as peels enclosing vegetative reproductive organs (bulb) such as garlic peels possess allelopathic substances that can inhibit or promote germination of neighboring plants (Grafi and Singiri 2022). Multiple reports have demonstrated selective effects of crop residues on germination and post germination growth of a variety of plant species. For example, glume extracts of *Aegilops kotschyi* and hull of *Avena fatua* inhibited germination of lettuce seeds (Waisel and Adler 1959; Chen et al. 1982), husk of winter wild oat (*Avena sterilis*) specifically inhibit seed germination of *Sinapis alba* but not of *Brassica juncea* (Raviv et al. 2020), glume extract of *Leymus chinensis* inhibited germination and root length of Chinese cabbage (Ma et al. 2010) and crop residues of *Sorghum* cultivars contain allelopathic substances that inhibit wheat seedling growth (Ben-Hammouda et al. 1995). Also, as shown here (Figs. 4 and 5), extract derived from garlic residues (garlic straw) strongly inhibited germination of the weed *A. palmeri* but had no effect on germination of *A. theophrasti*, yet it strongly inhibited post germination growth of both. Selective allelopathy (Kushima et al. 1998; Ohno et al. 2001) provides a means for reducing competition for resources (Evenari 1949) on the one hand, and on the other hand permitting seed germination of other species for facilitative plant-plant interaction (Brooker et al. 2008).

### Bacterial growth

Generally, microorganisms play an important role in the industry and are used for production of multiple substances and commodities including beverages (e.g., beer, wine), enzymes (e.g., proteases), antibiotics, vitamins, and organic acids (Ferreira et al. 2018; Lorenzo et al. 2018). The examined AIBWs displayed different effect on *S. aureus* growth with garlic straw having a very strong inhibitory effect similarly to the effect of the antibiotic kanamycin. The strong effect of garlic straw on bacterial growth is consistent with previous reports demonstrating the broad-spectrum activity of garlic clove extract on microbial growth (Harris et al. 2001; Curtis et al. 2004; Singiri et al. 2022). All WB extracts as well as Gp showed enhanced bacterial growth. We found that a medium mixture composed of 50% WB-1 and 50% LB strongly enhanced *S. aureus* growth in comparison to LB. This is consistent with previous reports showing that extracts derived from the husk of *Avena sterilis* and from pericarps of *Sinapis alba* and *Brassica juncea* promoted bacterial growth (Godwin et al. 2017; Raviv et al. 2020; Swetha et al. 2021). Since the cost of microbial growth medium is high often limiting the magnitude of production of beneficial substances and multiple commodities, AIBWs, like WB may offer a low-cost, sustainable, eco-friendly medium for growing microorganisms (Siddeeg et al., 2020; Lopes and Ligabue-Braun 2021; Sodhi et al. 2022).

## Conclusions

The data presented here together with a large body of published reports give further support to the idea of circular economy, a concept which is now developing rapidly. As mentioned above, AIBWs constitute a significant global issue that gets worse over time due to the lack of efficient ways to treat or utilize wastes (Wilson et al. 2015; Kaza et al. 2018). The limited reuse and recycling of AIBWs could be overcome, at least partly, by detailed analysis of AIBWs for their beneficial substances and activities that will allow their efficient sorting for various agro-industrial applications. The realization that each batch of AIBW is unique and its properties may be influenced by multiple factors, such as plant species and varieties, growth conditions, and milling processing, is of prime importance. As a result, every AIBW should be thoroughly examined to ensure that it is properly and efficiently sorted for agro-industrial applications. In-depth analysis should include metabolic and proteomic analysis of AIBW batches including specific analysis of growth factors (e.g., phytohormones), and untargeted metabolic analysis (primary metabolites and specialized metabolites*)* followed by direct analyses of their wide impact and particular effect on plant and microbial growth. The findings that WB and GSP contain notable amounts of proteases as well as strigolactones should encourage their utilization for various industrial applications (Troncoso et al. 2022), crop protection (Pacifico et al. 2021) and promote farmers to select wheat and garlic for intercropping and crop rotation or even using AIBWs directly as a soil amendment to combat root parasitic weeds (Aly and Dubey 2014; Fernández-Aparicio et al. 2011). WBs should be further examined for their capacity to serve as a bacterial growth medium and to enhance growth of microorganisms that are important for the agro-industrial sector. In conclusion, while AIBWs can be used as rich sources for purification of specific valuable components like proteases and phytohormones, they can be applied directly as extracts or raw material for diverse agro-industrial uses.

### Electronic supplementary material

Below is the link to the electronic supplementary material.

Supplementary Material 1 Supplementary Tables S1-S8

Supplementary Material 2 Supplementary Figures S1-S5

## Data Availability

Data will be made available on request.
