# Supplementary material for: Detailed analysis of agro-industrial byproducts/wastes to enable efficient sorting for various agro-industrial applications
Source: Bioresour Bioprocess. 2024 May 4;11(1):45. doi: 10.1186/s40643-024-00763-7 (PMC11069496; doi:10.1186/s40643-024-00763-7)
Supplement: Supplementary file 2 — Supplementary Material 2 Supplementary Figures S1-S5 [file 40643_2024_763_MOESM2_ESM.docx]

Supplementary materials

**Detailed analysis of agro-industrial byproducts/wastes to enable efficient sorting for various agro-industrial applications**

Govindegowda Priyanka^†^, Jeevan R. Singiri^†^, Zachor Adler-Agmon, Sasank Sannidhi, Spurthi Daida, Nurit Novoplansky and Gideon Grafi*

French Associates Institute for Agriculture and Biotechnology of Drylands, Jacob Blaustein Institutes for Desert Research, Ben-Gurion University of the Negev, Midreshet Ben Gurion 84990, Israel

**
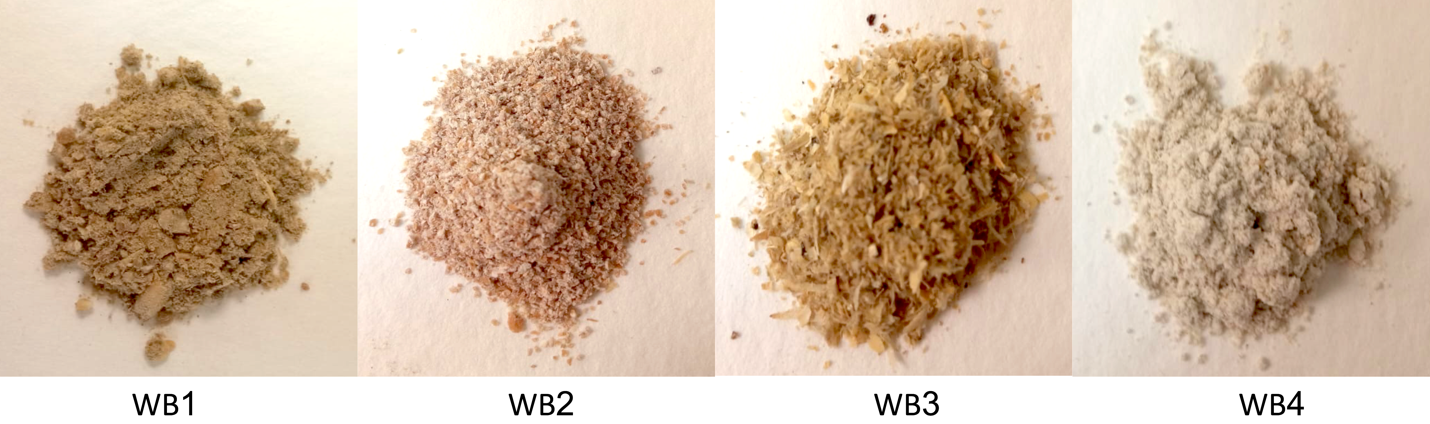
**

Fig. S1. Various batches of wheat bran (WB) obtained from a local milling company (Stibel, Beer Sheva, Israel) and used in the present study.


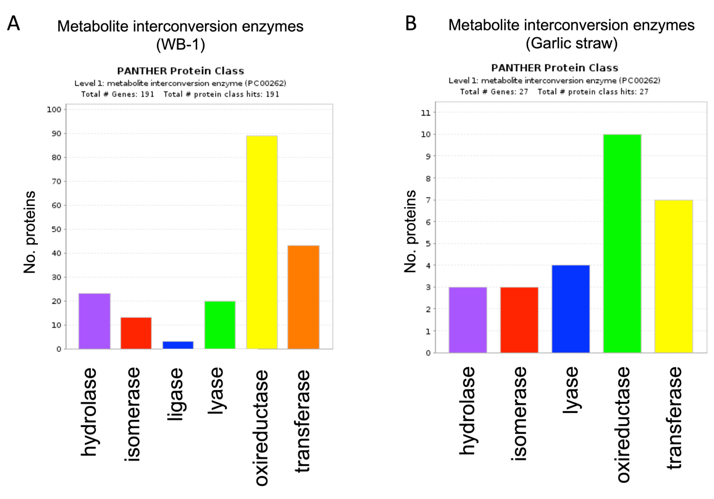


Fig. S2. Categorization of metabolite modifying enzymes identified in WB-1 (A) and in garlic straw (B). Categorization was performed with PANTHER v.16 (Mi et al., 2021).

Mi, H.; Ebert, D.; Muruganujan, A.; Mills, C.; Albou, L.P.; Mushayamaha, T.; Thomas, P.D. 2021. PANTHER version

16: a revised family classification, tree-based classification tool, enhancer regions and extensive API. *Nucleic Acids*

*Res*. *49*, D394-403.


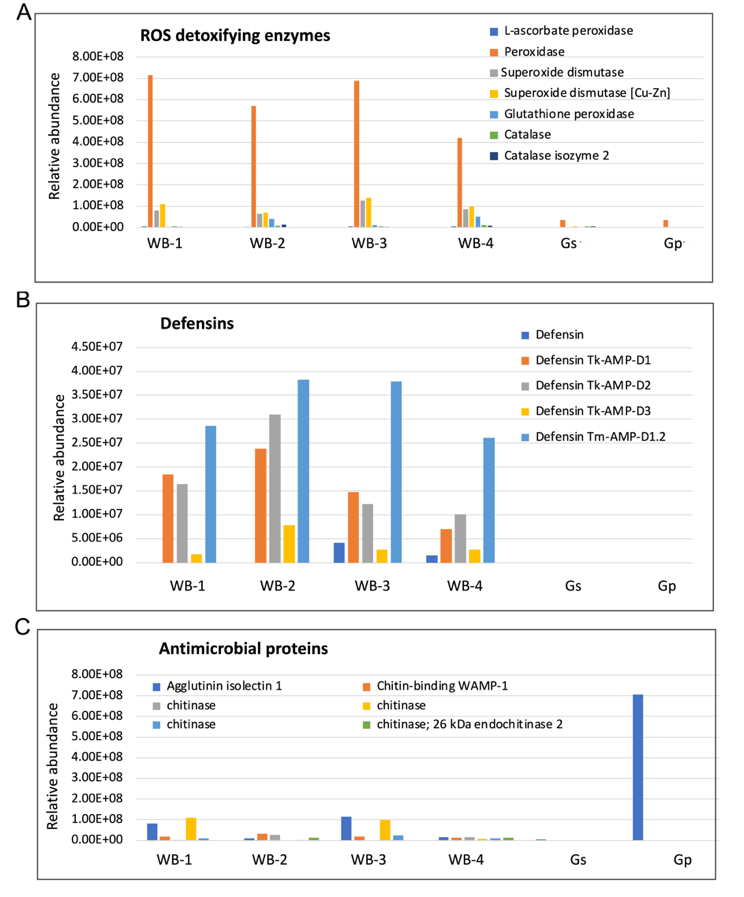


Fig. S3. The relative abundance of various protein groups in AIBWs. (A) Reactive oxygen species (ROS) detoxifying proteins. (B) Anti-fungal proteins, defensins. (C) Other antimicrobial proteins chitinases and agglutinin isolectin 1.


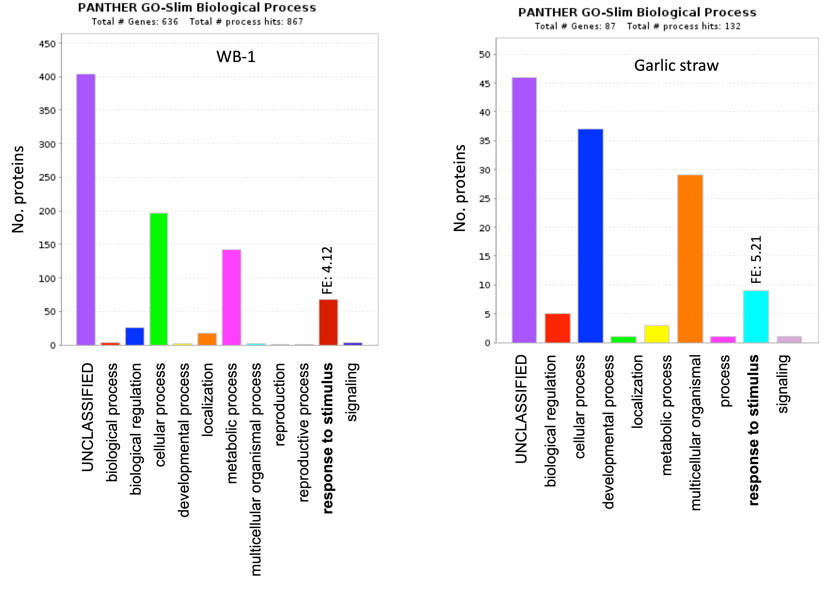


Fig. S4. Biological process categorization of proteins identified in WB-1 and Garlic straw (Gs). Note proteins implicated in response to stimulus are overrepresented in WB-1 and Gs. Fold enrichment (FE) is shown (FDR<0.05, PANTHER classification system).


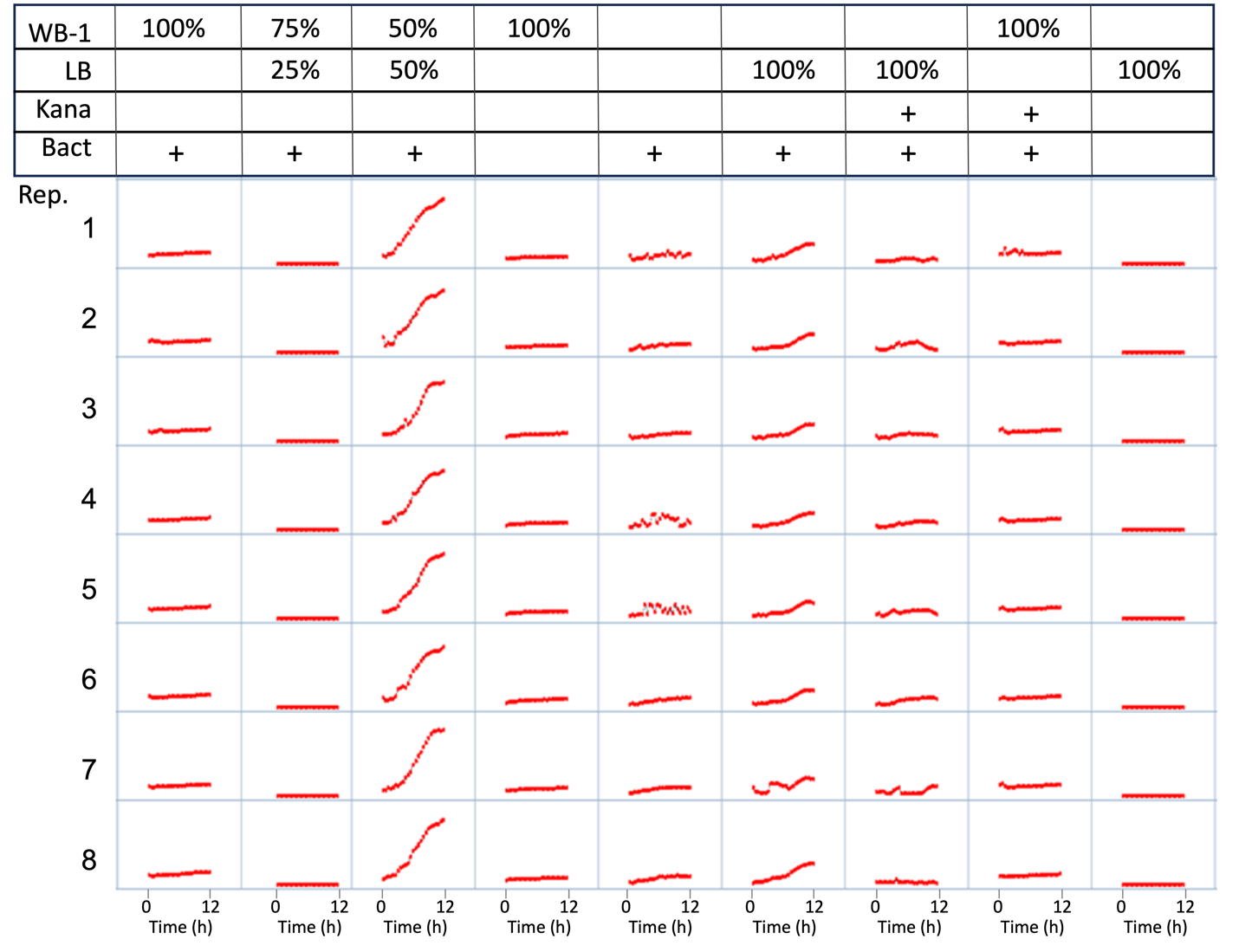


Fig. S5. WB-1 extract can serve as a partial substitute for bacterial growth medium to enhance bacterial growth. *S. aureus* (10 μl of overnight culture) was grown in a flat-bottom 96-well microtiter plate in the presence of various concentrations of WB-1 extract diluted with LB and compared to LB (100%). Bacterial growth experiments were performed in 8 replicates each treatment, Kanamycin (Kana) was used as an antibiotic reference. Bacterial growth (OD_595_) was measured at 0.5 h intervals in the course of 12 h.
